# Quercetin Combined With Human Umbilical Cord Mesenchymal Stem Cells Regulated Tumour Necrosis Factor-α/Interferon-γ-Stimulated Peripheral Blood Mononuclear Cells *via* Activation of Toll-Like Receptor 3 Signalling

**DOI:** 10.3389/fphar.2020.00499

**Published:** 2020-04-24

**Authors:** Guiling Chen, Yang Ye, Ming Cheng, Yi Tao, Kejun Zhang, Qiong Huang, Jingwen Deng, Danni Yao, Chuanjian Lu, Yu Huang

**Affiliations:** ^1^ Second Affiliated Hospital, Guangzhou University of Chinese Medicine, Guangzhou, China; ^2^ Department of National Institute of Stem Cell Clinical Research, Guangdong Provincial Hospital of Chinese Medicine, Guangzhou, China; ^3^ Guangdong Provincial Academy of Chinese Medical Sciences, Guangzhou, China; ^4^ Department of Shanghai Zhangjiang Biobank, National Engineering Centre for Biochip at Shanghai, Shanghai, China; ^5^ Department of Dermatology, Guangdong Provincial Hospital of Chinese Medicine, Guangzhou, China

**Keywords:** rheumatoid arthritis, quercetin, human umbilical cord mesenchymal stem cell, Toll-like receptor 3, interleukin 6, indoleamine 2,3-dioxygenase

## Abstract

The beneficial effect of quercetin in rheumatic diseases is unclear. Studies have already confirmed that human umbilical cord mesenchymal stem cells (hUCMSCs) alleviate some symptoms of rheumatoid arthritis (RA) by their immunosuppressive capacities. This study explored whether there are additive effects of quercetin and hUCMSCs on peripheral blood mononuclear cells (PBMCs) under simulated rheumatic conditions. hUCMSCs were pretreated with quercetin (10 μM) before coculture with TNF-α/IFN-γ-stimulated PBMCs at a ratio of 1:1 for 3 days. PBMC proliferation was inhibited, and the proportion of Th17 cells was shifted. These effects may be related to the effect of quercetin on functional molecules in hUCMSCs, including nitric oxide (NO), indoleamine 2,3-dioxygenase (IDO), interleukin 6 (IL-6) and Toll-like receptor-3 (TLR-3) and the Akt/IκB pathways. These results suggest that quercetin effectively promoted the immunoregulatory effect of hUCMSCs by inhibiting the Akt/IκB pathway, activating the Toll-like receptor-3 pathway, and regulating downstream cytokines.

## Introduction

Rheumatoid arthritis (RA) is a long-term autoimmune disorder involving multiple systems. RA is characterized by the destruction of cartilage and bone by inflammatory cytokines, such as tumour necrosis factor-α (TNF-α) and interferon-γ (IFN-γ) (McInnes and Schett, 2011; Lubberts, 2015), and involves the complex interaction of T helper 1 cells (Th1 cells), Th17 cells, Th2 cells, immunoregulatory cells (such as Treg cells) and monocytes/macrophages. Compared to therapeutic agents and disease-modifying antirheumatic drugs (DMARDS), biological agents, such as TNF inhibitors, are efficacious and safe for the management of arthritis. Mesenchymal stem cells (MSCs), which are multipotent progenitor cells, are emerging as a promising biological agent for treating immune diseases. MSCs can be isolated from bone marrow (BM), umbilical cord, placenta, and adipose tissue, and these cells have the ability to differentiate into various other mesodermal cell lineages, including chondrocytes, adipocytes, and osteoblasts. Another property of MSCs is their anti-inflammatory and immunosuppressive effects. A large number of preclinical studies (Alunno et al., 2015; Yang et al., 2016; Zhang et al., 2017) and clinical studies (Dahbour and Jamali, 2017; Riordan et al., 2018; Zhang et al., 2018) have shown that MSCs considerably alleviate symptoms of autoimmune diseases. Compared with stem cells from other sources, umbilical cord mesenchymal stem cells possess advantages, including rapid, non-invasive harvest procedures, easy expansion *in vitro*, and ethical access; additionally, umbilical cord mesenchymal stem cells exhibit robust immunosuppressive functions (Jin et al., 2013; Wang et al., 2016).

The flavonoid quercetin is widely distributed in plants, foods, and beverages and is an active ingredient that is abundant in traditional Chinese medicines, such as Huangqi Guizhi Wuwu tang. It is a classic formula for treating autoimmune diseases with clinical efficacy that is based on decades of clinical observation and clinical practice. The formula is composed of 5 herbs: *Astragalus mongholicus* Bge (Huangqi), *Cassia twig* (Guizhi), *Paeonia lactiflora* Pall. (Shaoyao), *Zingiber officinale* Roscoe (Shengjiang), and *Ziziphus jujuba* Mill. (Dazao). **Pang et al. conducted a meta-analysis of sixteen randomized controlled trials with a total of 1,173 patients suffering diabetic peripheral neuropathy. The results revealed that Huangqi Guizhi Wuwu tang had significant therapeutic efficacy in treating peripheral diabetic neuropathy (Pang et al., 2016). Previous studies have shown that quercetin has various biological actions, such as antioxidative and anti-inflammatory effects (Chen et al., 2018), including inhibiting inflammatory cytokines, such as ROS, IFN-γ, TNF-α, and IL-2 (Meng et al., 2018). Quercetin suppresses the secretion of inflammatory cytokines by regulating transcription factors (NF-κB) (Chen et al., 2005; Potapovich et al., 2011).

We hypothesized that quercetin has an anti-inflammatory effect and an additive effect when combined with UCMSCs, and this study aimed to investigate the mechanism by which quercetin and UCMSCs affect TNF-α and IFN-γ-stimulated PBMCs and explain the effect of quercetin and UCMSC treatment on clinical symptoms and disease activity in patients with RA.

## Materials and Methods

### Isolation and Culture of UCMSCs

Human umbilical cords (n = 3) were isolated from tissue obtained after full-term, healthy births. The mothers had been informed beforehand and consented to donate. In brief, the vein and artery of the UC were removed to retain Wharton’s jelly. Wharton’s jelly was cut into small pieces (1–2 mm^3^) and placed into 100 mm tissue culture dishes (NUNC, Thermo Fisher Scientific, USA). A total of 10 ml complete culture medium containing MEM-α (HyClone, USA) supplemented with 5% serum substitute (UltraGRO Advanced, HELIOS, USA) was added to the tissue culture dishes and incubated at 37°C in a 5% carbon dioxide incubator for 7 to 8 days. The medium was changed every 3 to 4 days. After 7 to 8 days, the tissue pieces were removed, and the cells that had attached to the tissue culture dishes were cultured. After another 7 to 8 days, the cells reached 50% to 60% confluence and were passaged into a T175 culture flask at a density of 2×10^6^/cm^2^. Each flask contained 25 ml of MEM-α supplemented with 5% serum substitute (UltraGRO Advanced, HELIOS, USA) and maintained at 37°C in a 5% carbon dioxide incubator. UCMSCs were further passaged when they reached 90% confluence. All UCMSCs used in this study were used within passages 3–5. This study was approved by the Ethics Committee at Guangdong Provincial Hospital of Chinese Medicine and was conducted in accordance with the 1989 Declaration of Helsinki.

### UCMSC Characterization

UCMSCs (1 × 10^6^ per tube) were phenotypically characterized by flow cytometry (FACS Aria II, BD Biosciences, USA) using the following antibodies: CD105-PerCy, CD90-FITC, CD44-PE, CD73-FITC, and a negative MSC cocktail (CD45/CD34/CD11b/CD19/HLA-DR) (BD Biosciences, USA). A total of 20,000 events were recorded for each sample, and the data were analysed using FlowJo software (BD Biosciences, USA). In addition, UCMSCs were functionally characterized by multipotent differentiation to adipocytes, osteocytes, and chondrocytes using differentiation and staining kits (Biological Industries, Israel). Briefly, 6×10^4^ cells/well were seeded in 24-well plates for adipogenic or osteogenic differentiation assays, and 1×10^5^ cells/well were seeded in 96-well U-bottom culture plates for chondrogenic differentiation assays using complete culture medium. All plates were placed in a 5% carbon dioxide incubator at 37°C for 24 to 48 h, and when the cells were 80% to 90% confluent, the medium was changed to differentiation medium (05-330-1-1B, 05-331-1-01 & 05-332-1-15 for adipogenesis; 05-442-1 for osteogenesis; 05-220-1B & D for chondrogenesis). Then, the cells were incubated for 10 to 21 days, and the differentiation medium was changed every 3 to 4 days. Oil red O staining, Alizarin red staining, and Alcian blue staining were used to evaluate adipogenesis, osteogenesis, and chondrogenesis.

### Cell Viability Measurement

The effect of quercetin (HPLC≥98%, China National Analytical Centre, Guangzhou (NACC)) on UCMSC viability was assessed by measuring the absorbance of 3-(4.5- dimethylthiazol-2-yl)-2,5-diphenyl tetrazolium bromide (MTT) dye in the cells. For this, 2 × 10^3^ UCMSCs were cultured in 96-well culture plates and then treated with quercetin (0, 1.25 μM, 2.5 μM, 5 μM, 10 μM, and 20 μM) for 3 days. Subsequently, 10 μl of MTT (5 mg/ml) was added to each well, and the plates were incubated at 37°C for 4 h. Then, the MTT and medium were discarded and replaced with DMSO, and the plates were incubated for 10 min. The optical density was read on a PerkinElmer VICTOR X5 (PerkinElmer, USA) with a monochromatic microplate reader at a wavelength of 570 nm.

### Isolation and Activation of PBMCs

PBMCs were isolated from the blood of healthy volunteers by centrifugation using Lymphoprep (Axis-Shied, Norway) and washed three times with PBS. Isolated PBMCs were then activated with 20 ng/ml TNF-α and 50 ng/ml IFN-γ (PeproTech, USA) and used in coculture experiments.

### Immunosuppression Assays

To test the effect of UCMSCs in combination with quercetin on PBMC proliferation, 5 × 10^5^ UCMSCs were cocultured for 3 days in 6-well plate**s** with carboxyfluorescein succinimidyl ester CFSE-labeled (Beyotime, China) PBMCs (1:1 ratio) that were activated with TNF-α and IFN-γ; quercetin (10 μM) was added to the UCMSCs 2 h before coculture of the UCMSCs with the PBMCs. On the third day, the PBMCs were collected, and PBMC proliferation was analysed by flow cytometry.

The presence of Th1, Th2, and Th17 cells was determined by measuring IFN-γ-FITC, IL-4-PE, and IL-17-PE cells using flow cytometry. UCMSCs (5 × 10^5^) were cultured in 6-well plate**s** with 20 ng/ml TNF-α, 50 ng/ml IFN-γ, and 15 ng/ml brefeldin A (added 3 h after TNF-α and IFN-γ) (all from PeproTech, USA) and then cocultured with 5 × 10^5^ UCMSCs (1:1 ratio) that were treated or without 10 μM quercetin for 72 h at 37°C. After incubation with FITC-conjugated anti-human CD3 and PE-Cyanine7-CD4 antibodies (eBioscience, USA), the cells were fixed and permeabilized and further incubated with FITC-conjugated anti-human IFN-γ (eBioscience, USA), phycoerythrin (PE)-conjugated anti-human IL-4 (eBioscience, USA), and PE-conjugated anti-human IL-17 (eBioscience, USA) antibodies. The cells were then resuspended in PBS and subjected to flow cytometric analysis.

### Cytokine Examination

The levels of the cytokines IL-6 and IL-10 in the supernatant were evaluated by enzyme-linked immunosorbent assay (ELISA) using kits purchased from DAKEWE (Shenzhen, China). Tests were performed according to the manufacturer’s recommended protocols. Each cytokine standard and sample was run in duplicate.

### Nitrite Measurement

Nitrite released into the medium was used as a measure of NO production. Nitrite determination was performed using Griess reagent (Invitrogen, Thermo Fisher Scientific, USA) according to the manufacturer protocols.

### qRT-PCR

Total RNA from UCMSCs was extracted in the presence or absence of PBMCs and/or quercetin with a FastPure Cell/Tissue total RNA isolation kit (Vazyme, China). RNA concentration and purity were estimated by optical density measurement. RNA isolation was followed by DNase digestion. Total RNA (1 μg) was reverse transcribed to cDNA with a HiScript III RT SuperMix for qPCR kit (Vazyme, China) at 37°C for 15 min and 85°C for 5 s. Quantitative PCR was performed using ChamQ Universal SYBR qPCR master mix (Vazyme, Q711-02) and an Applied Biosystems QuantStudio 3 Detection System according to the manufacturer’s recommendations (Thermo Fisher Scientific, USA). Specific primers for IDO were designed using Primer3 software (**Table 1**). Expression levels of the transcripts were normalized to the housekeeping gene glyceraldehyde-3-phosphate dehydrogenase (GAPDH). For quantification, the values are expressed as the relative mRNA level of a specific gene as determined using the 2−ΔCt method.

**Table 1:** Primer sequences and access numbers of study genes.

Gene Name	Forward Primer (5′ to 3′)	Reverse Primer (5′ to 3′)
GAPDH	ACAACTTTGGTATCGTGGAAGG	GCCATCACGCCACAGTTTC
IDO	TCTCACAGACCACAAGTCACAGC	AGAGTTGGCAGTAAGGAACAGCA

### Western Blot Analysis

UCMSCs were washed with PBS once and then collected. Subsequently, the cells were lysed in RIPA buffer (Beyotime Biotechnology, China) at 4°C for 30 min. Then, the mixture was centrifuged at 16,000 rcf for 20 min. The lysates were incubated at 95°C for 5 min, separated by 10% SDS-PAGE (Beyotime Biotechnology, China) and transferred to a polyvinylidene difluoride (PVDF) membrane (Millipore, USA). Each PVDF membrane was blocked in TBST with 5% nonfat milk or BSA with gentle shaking for 60 min and then incubated with their respective primary monoclonal antibodies (1:1000; diluted in primary antibody solutions, purchased from Beyotime Biotechnology) against p-IκB (2859), p-AKT (4060), AKT (4691), Toll-like receptor 3 (6961), and GAPDH (5174) (Cell Signalling Technology, USA) overnight at 4°C. Then, each PVDF membrane was incubated with 1:5000 horseradish peroxidase-conjugated secondary antibody (BA1054) (BOSTER, China) in TBST with 5% nonfat milk or BSA for 1 h. Finally, the membranes were visualized by enhanced chemiluminescence staining. The intensities of the bands were quantified with a computerized densitometer.

### Statistical Analysis

Statistical analysis was performed using SPSS 13.0. The data are expressed as the means ± SEM. One-way ANOVA was used to analyse the data. Significance was set at *P* < 0.05.

## Results

### Isolation and Characterization of UCMSCs

UCMSCs were grown from the tissue for 5 to 7 days, and single cell-derived clones gradually reached confluence with a whirlpool-like arrangement. When cells were 50% to 60% confluent in 100 mm culture dishes, the cells were passaged into a T175 culture flask at a density of 2×10^6^/cm^2^. The cells were digested and passaged into another T175 culture flask when they reached 80% to 90% confluence.

**Figure 1:**
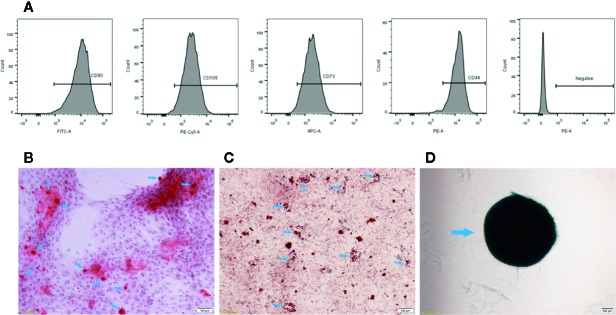
Characterization of UCMSCs. **(A)** Representative flow cytometry histograms showing high expression levels of CD105, CD90, CD44, and CD73 and low expression levels of the negative MSC cocktail (CD45/CD34/CD11b/CD19/HLA-DR) on the surface of UCMSCs. **(B)** Osteogenesis. Red calcified nodules formed after 7 to 10 days of osteogenic induction (Alizarin red staining, see arrows). **(C)** Adipogenesis. Orange red lipid droplets in the cytoplasm after 21 days of adipogenic induction (oil red O staining, see arrows). **(D)** Chondrogenesis. Proteoglycan aggrecan in mature chondrocytes was stained blue after 21 days of chondrogenic induction (Alcian blue staining, see arrows). Scale bar = 100 μm.

Cultured UCMSCs that were isolated from human umbilical cords showed the capacity for differentiation into osteoblasts, adipocytes, and chondrocytes after culture in differentiation medium for 10 to 21 days. The formation of lipid droplets in mature adipocytes and mineralized calcified nodules in osteoblasts and chondrocyte clusters were observed. Calcified nodules in osteoblasts were stained red using Alizarin red (**Figure 1B**), lipid droplets in mature adipocytes were stained orange using oil red O (**Figure 1C**), and the proteoglycan aggrecan in chondrocytes was stained blue using Alcian blue (**Figure 1D**).

In addition, UCMSCs presented a typical MSC immunophenotype, with positive expression of CD105 (97.57% ± 0.67%), CD73 (97.83% ± 0.51%), CD44 (95.37% ± 1.13%), and CD90 (99% ± 0.7%), and the cells lacked CD34, CD45, and HLA-DR (total 0.1% ± 0.1%) markers (**Figure 1A**).

### Quercetin Did Not Influence UCMSC Viability, Morphology, Phenotype or Cell Cycle

**Figure 2:**
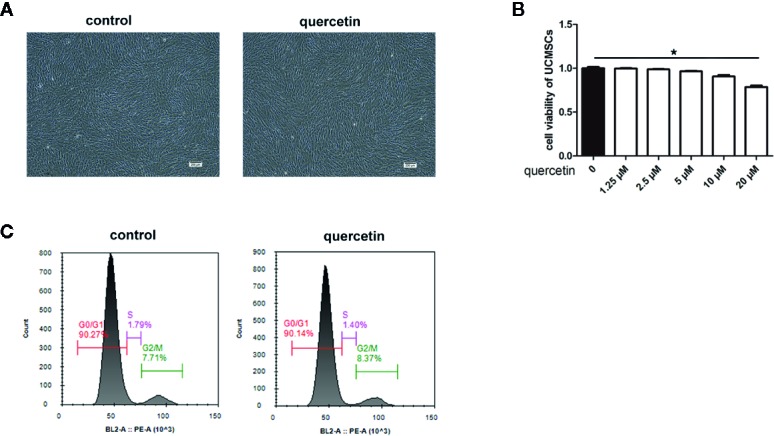
Morphology, viability, phenotype, and cell cycle of UCMSCs. Untreated UCMSCs and UCMSCs that were pretreated with 10 μM quercetin were cultured for 3 days. **(A)** Morphology (100×). Untreated UCMSCs and UCMSCs that were pretreated with 10 μM quercetin shared the same morphology. **(B)** UCMSC viability was examined by MTT assay. Quercetin at 20 μM influenced UCMSC viability. *P < 0.05, mean ± SEM, n=5. **(C)** The proportion of cells in the G1, S, and G2/M phases of the cell cycle in untreated UCMSCs and UCMSCs that were pretreated with 10 μM quercetin. No significant differences were detected. Representative examples are shown.

The effect of quercetin on the basic properties of UCMSCs, including viability, morphology, surface marker expression, and cell cycle, was evaluated. No changes were observed in UCMSC viability in an MTT assay after 3 days of culture in complete medium or with 1.25 μM, 2.5 μM, 5 μM, and 10 μM quercetin (**Figure 2B**). In addition, 20 μM quercetin inhibited UCMSC viability, and a dose of 10 μM quercetin was used in the subsequent study. Regarding morphology and phenotype, UCMSCs treated with 10 μM quercetin and untreated UCMSCs shared similar shapes (**Figure 2A**) and the same surface markers, and the cells were positive for CD44, CD73, CD90, and CD105 and negative for CD34, CD45, CD19, CD11b, and HLA-DR (data not shown). Both of them had analogous proportions of cells in the G1, S, and G2/M phases of the cell cycle (**Figure 2C**).

### Quercetin Enhanced UCMSC Immunosuppression of PBMCs

**Figure 3:**
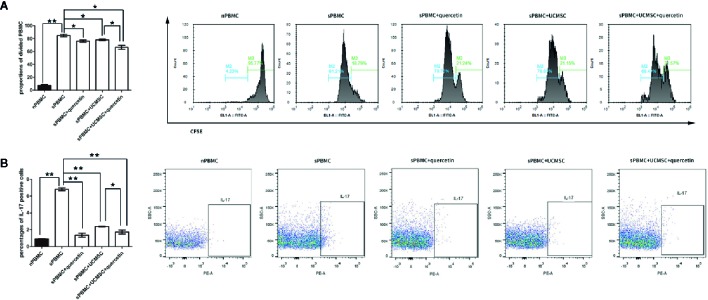
Quercetin enhanced UCMSC immunomodulation. UCMSCs were cocultured with TNF-α/IFN-γ-activated PBMCs (1:1 ratio) for 3 days. **(A)** CFSE-labeled PBMCs were cocultured with UCMSCs that were treated with or without quercetin for 3 days, and PBMC proliferation was determined by the proportions of attenuated CFSE-fluorescent cells (divided cells). Both UCMSCs and quercetin suppressed PBMC division, and UCMSCs and quercetin acted on PBMCs additively; they had a combined inhibitory effect on PBMC division. Mean ± SEM, n = 3, *P < 0.05, ** *P* < 0.01. **(B)** Th17 cells were measured by IL-17-positive cells from purified CD4+ T cells. Quercetin and UCMSCs alone or in combination inhibited the proliferation of Th17 cells, and quercetin enhanced the suppressive effect of UCMSCs when UCMSCs were pretreated before coculture. Mean ± SEM, n=3, *P < 0.05, ** P < 0.01.

The inhibitory effects of UCMSCs on PBMC proliferation were evaluated, and the difference between UCMSCs with and without quercetin treatment was compared. CFSE-labeled PBMCs were collected and detected by FACS on the third day after coculture with TNF-α/IFN-γ-activated UCMSCs that were treated with or without 10 μM quercetin. The data showed that the proportion of divided PBMCs in the stimulated group (sPBMCs) reached 84.74% ± 1.85% (M2), while the proportion of unstimulated PBMCs (nPBMCs) was 7.42% ± 1.6% (M2). UCMSCs significantly inhibited the proliferation of PBMCs in the coculture system, showing 78.05% ± 1.41% (M2) divided cells, and quercetin (66.45% ± 2.88% (M2)) enhanced the effect. As shown in **Figure 3A**, when UCMSCs were treated with quercetin before coculture, the division rates of PBMCs after TNF-α/IFN-γ activation decreased significantly. Th17 subsets in PBMCs were evaluated by counting the CD4^+^IL-17^+^ cells using flow cytometry. The proliferation of CD4^+^IL-17^+^ cells was promoted in stimulated PBMCs but inhibited in activated PBMCs that were cocultured with UCMSCs compared to stimulated PBMCs, and the addition of quercetin to the coculture system had a combined inhibitory effect on CD4^+^IL-17^+^ cell proliferation (**Figure 3B**).

These results indicated that the suppressive effects of UCMSCs on PBMC proliferation were augmented by quercetin treatment.

### Quercetin Promoted UCMSCs to Express TLR-3 and Suppressed AKT or IκB

**Figure 4:**
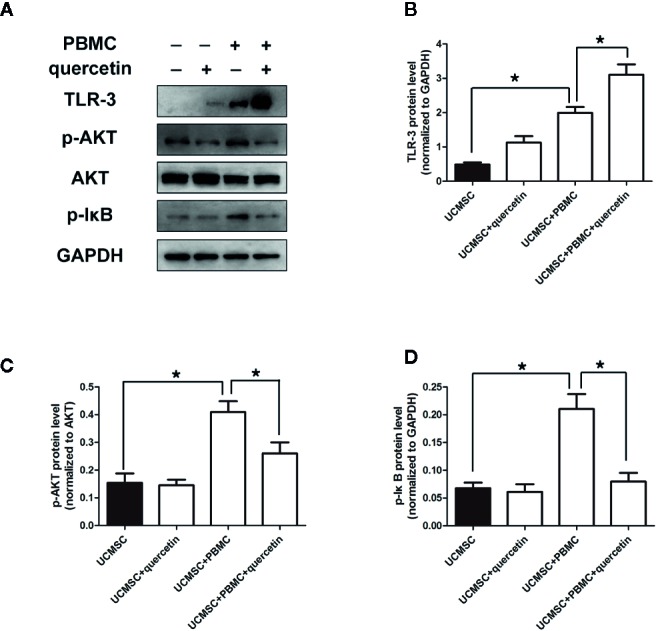
Changes in signaling pathways in UCMSCs. UCMSCs were cocultured with TNF-α/IFN-γ-activated PBMCs (1:1 ratio) in the presence or absence of quercetin for 3 days. **(A)** Expression of TLR-3 and AKT/IκB was assessed by Western blotting. Quercetin induced TLR-3 expression and inhibited AKT/IκB expression in UCMSCs. **(B**–**D)** The bar graphs show the quantification of the indicated proteins. Mean ± SEM, n = 3. *P < 0.05.

To explore the mechanism by which quercetin and UCMSCs suppressed PBMC proliferation, the expression of TLR-3, AKT, and IκB was assessed in untreated UCMSCs, as well as in UCMSCs that were treated with 10 μM quercetin or/and activated PBMCs for 3 days. TLR-3 is linked to MSC immunosuppressive capacity, promoting MSCs to secrete cytokines that inhibit the inflammatory response. AKT or IκB are signals that are relevant to inflammatory reactions. The data showed that UCMSCs overexpressed TLR-3 when cocultured with activated PBMCs, and quercetin enhanced this effect. As shown in **Figure 4A**, UCMSCs alone expressed low levels of TLR-3. When cocultured with activated PBMCs, UCMSCs showed increased expression of TLR-3, and when UCMSCs were pretreated with quercetin in the coculture system, TLR-3 expression was higher. p-AKT and p-IκB expression was increased in UCMSCs that were cocultured with stimulated PBMCs, and quercetin decreased p-AKT and p-IκB expression (**Figures 4A–D)** show the quantification of the TLR-3,p- AKT and p- IkB.

### Quercetin Promoted UCMSCs to Produce the Anti-Inflammatory Factors IL-6, NO, and IDO in Response to Activated PBMCs

**Figure 5:**
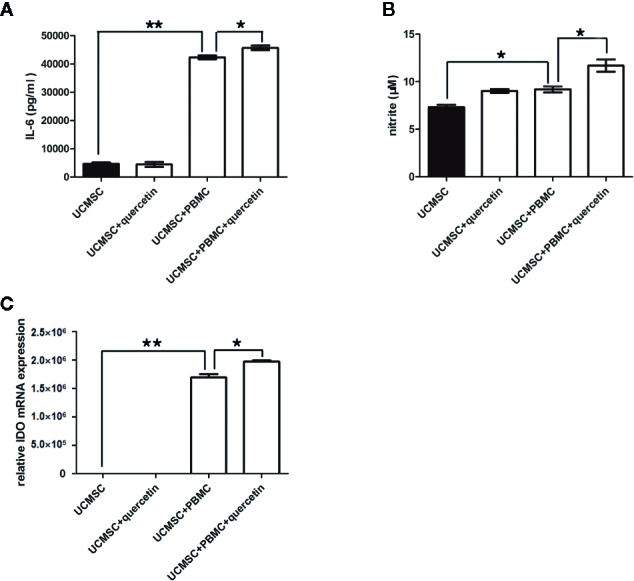
Changes in anti-inflammatory molecules in UCMSCs. TNF-α/IFN-γ-activated PBMCs (1:1 ratio) in the presence or absence of quercetin for 3 days. The anti-inflammatory molecules IL-6 **(A)**, NO **(B)**, and IDO **(C)** were determined by ELISA, Griess assay and RT-PCR.. UCMSCs overexpressed IL-6, NO, and IDO in the coculture system with PBMCs. Quercetin enhanced the effect. Mean ± SEM, n = 3. *P < 0.05, ** *P* < 0.01.

Furthermore, the levels of the anti-inflammatory factors IL-6, NO, and IDO, which are linked to TLR-3 signaling, were assessed in UCMSCs that were pretreated with or without 10-μM quercetin and co-cultured with activated PBMCs for 3 days. As shown in **Figures 5A, B**, IL-6 and NO were increased in the supernatant of UCMSCs in the coculture system, and 10 μM quercetin enhanced the effect, with higher levels of IL-6 and NO. Consistently, IDO transcripts were significantly increased in UCMSCs that were treated with activated PBMCs. Quercetin increased the level of IDO transcripts in UCMSCs in the coculture system (**Figure 5C**). IL-10 expression was not affected by quercetin or activated PBMCs (data not shown).

## Discussion

Rheumatoid arthritis is a systemic inflammatory autoimmune disease that is characterized by synovitis. Infiltrated T cells (Th1 cells, Th17 cells, and Th2 cells), resident macrophages, and Treg cells and the overproduction of inflammatory factors (such as TNF-α, IFN-γ, IL-6, and IL-17) have been extensively studied to explain the mechanism. Their cross-interaction causes complex inflammatory cascades all lead to persistent synovial inflammation, and associated damage to articular cartilage and underlying bone.

MSCs are emerging as a new type of immunosuppressant in multiple autoimmune diseases. Biological agents are non-dependent, non-resistant, and have no side effects that are increasingly favoured by clinical researchers. How to improve the immunosuppressive effect of MSCs is currently a concern. Quercetin, a traditional Chinese medicine monomer, has anti-inflammatory and antioxidant effects. A few studies have focused on its anti-inflammatory effects on autoimmune diseases (Milenkovic et al., 2010; Wang et al., 2013; Javadi et al., 2017).

Consistent with other studies (Allanore et al., 2001; Kim et al., 2007; Xiong et al., 2014), this study used TNF-α/IFN-γ to mimic the *in vitro* condition of RA, which promoted PBMC proliferation and amplified Th17 subsets. Quercetin and UCMSCs were then administered to stimulate the PBMCs. The results showed that quercetin and UCMSCs both inhibited the inflammatory response. They had an additive effect on activated PBMCs. Notably, quercetin alone did not affect the proliferation, phenotype, or cell cycle of UCMSCs.

Furthermore, the results demonstrated that quercetin downregulated p-AKT/p-IκB expression and upregulated TLR-3 on UCMSCs and induced high levels of IL-6, IDO, and NO, which may explain some of the mechanisms by which UCMSCs suppress PBMC responses. Activation of the AKT/IκB pathway is involved in the inflammatory response in many studies (Burris et al., 2014; Ye et al., 2017; Harikrishnan and Jantan, 2018), and the findings of this study are consistent with these observations. TNF-α/IFN-γ stimulated PBMCs to activate p-AKT/p-IκB in cocultured UCMSCs; however, quercetin reversed these effects. TLR-3 is capable of polarizing MSCs towards an anti-inflammatory phenotype with enhanced immunosuppressive capacity (Opitz et al., 2009; Waterman et al., 2010; Cassano et al., 2018). According to the data, quercetin treatment of UCMSCs induced TLR-3 signaling and boosted the capacity of these cells to control PBMC inflammatory reactions. Quercetin alone has a direct suppressive effect. Poly(I:C) (a TLR-3 agonist) interacts with TLR-3 and prominently induces IL-6, IL-10, IL-11, LIF, VEGF, SDF1, and PGE2 (Mastri et al., 2012; Kim et al., 2018) in MSCs, which suggests that expression of the anti-inflammatory factors IDO, NO, and IL-6 by UCMSCs may be regulated by TLR-3.

Collectively, this study confirmed that UCMSCs combined with 10 μM quercetin ameliorated proliferation of TNF-α/IFN-γ-activated PBMCs by inducing TLR-3 signaling, providing an understanding of the mechanism by which quercetin potentiates UCMSCs and induces anti-inflammatory proteins in UCMSCs. This work may provide new ideas for the treatment of rheumatoid arthritis in the clinic.

## Data Availability Statement

The data generated for this study can be found in NCBI using the accession number NM_002164 (version NM_002164.6).

## Ethics Statement

The studies involving human participants were reviewed and approved by the ethics committee of Guangdong Provincial Hospital of Chinese Medicine. The patients/participants provided their written informed consent to participate in this study.

## Author Contributions

GC designed and conducted the study. KZ and QH conducted part of the study. YY, MC, and YT analyzed the data and interpreted the results. JD and DY polished the manuscript. CL provided the technical support and advices for the study. YH supervised the study. All authors contributed to the review and the approval of the final manuscript.

## Funding

This study was approved by grants from Special Funding for TCM Science and Technology Research of Guangdong Provincial Hospital of Chinese Medicine (project number: No.CMR-20170220-1001) and received grant from Special Funding for TCM Science and Technology Research of Guangdong Provincial Hospital of Chinese Medicine (project number: YN2016QJ02), the Traditional Chinese Medicine Bureau Foundation of Guangdong Province (project number:No.20183005).

## Conflict of Interest

The authors declare that the research was conducted in the absence of any commercial or financial relationships that could be construed as a potential conflict of interest.
